# Effect of SGLT2 inhibitors versus DPP4 inhibitors on major adverse kidney events in diabetic people with varied kidney function decline

**DOI:** 10.3389/fendo.2025.1647342

**Published:** 2026-01-15

**Authors:** Yu-Wen Cheng, Yi-Wei Kao, Shao-Wei Chen, Yi-Hsin Chan, Tze-Fan Chao

**Affiliations:** 1 The Cardiovascular Department, Chang Gung Memorial Hospital, Taoyuan, Taiwan; 2 College of Medicine, Chang Gung University, Taoyuan, Taiwan; 3 Department of Applied Statistics and Information Science, Ming Chuan University, Taoyuan, Taiwan; 4 Artificial Intelligence Development Center, Fu Jen Catholic University, Taipei, Taiwan; 5 Division of Thoracic and Cardiovascular Surgery, Department of Surgery, Chang Gung Memorial Hospital, Linkou Medical Center, Chang Gung University, Taoyuan, Taiwan; 6 Center for Big Data Analytics and Statistics, Chang Gung Memorial Hospital, Taoyuan, Taiwan; 7 School of Traditional Chinese Medicine, College of Medicine, Chang-Gung University, Taoyuan, Taiwan; 8 Microscopy Core Laboratory, Chang Gung Memorial Hospital, Taoyuan, Taiwan; 9 Division of Cardiology, Department of Medicine, Taipei Veterans General Hospital, Taipei, Taiwan; 10 Institute of Clinical Medicine, Cardiovascular Research Center, National Yang Ming Chiao Tung University, Taipei, Taiwan

**Keywords:** sodium-glucose cotransporter 2 inhibitors, dipeptidyl peptidase-4 inhibitors, type 2 diabetes, estimated glomerular filtration rate, acute kidney injury

## Abstract

**Introduction:**

The comparative kidney-protective effects of sodium-glucose cotransporter 2 inhibitors (SGLT2is) versus dipeptidyl peptidase-4 inhibitors (DPP4is) in people with type 2 diabetes (T2D) with varying past estimated glomerular filtration rate (eGFR) decline rates remain unclear.

**Methods:**

This retrospective study analyzed 4,011 propensity score-matched T2D people from a multi-center database, each with at least 2 years of eGFR data before therapy and 1 year of follow-up. The patients received either SGLT2i or DPP4i between June 2016 and December 2021.

**Results:**

Among paired patients, 23.7% (SGLT2i) and 25.4% (DPP4i) were rapid decliners (≥5 mL/min/1.73 m²/year). SGLT2i treatment was consistently associated with a slower eGFR decline than DPP4i, regardless of past eGFR slope. Post-treatment rapid eGFR decline decreased in both groups but remained higher in DPP4i users (20.5% vs. 15.4%). Those patients with past rapid eGFR decline receiving DPP4i rather than receiving SGLT2i remained at a higher risk for major adverse kidney events (MAKE) (a sustained 50% reduction in follow-up eGFR or the development of ESKD) and post-treatment rapid eGFR decline. Compared to DPP4i, SGLT2i therapy overall was associated with lower risks of MAKE (HR: 0.77; [95% CI: 0.64–0.94]), abrupt kidney function decline (HR: 0.76; [95% CI: 0.60–0.97]), and persistent rapid eGFR decline (HR: 0.76; [95% CI: 0.68–0.84]), with treatment benefits across different past eGFR decline categories. No difference in urinary albumin-to-creatinine ratio deterioration was observed between groups. The treatment benefits of SGLT2i over DPP4i were consistent across varying past eGFR slopes examined as a continuous variable.

**Conclusions:**

SGLT2i therapy was associated with better kidney outcomes and slower eGFR decline than DPP4i regardless of prior rapid eGFR decline.

## Introduction

Type 2 diabetes mellitus (T2D) is a major global health concern that markedly increases the risk of cardiovascular disease, microvascular complications, and chronic kidney disease (CKD) (1, 2). CKD, defined as the estimated glomerular filtration rate (eGFR) <60 mL/min/1.73 m² or the presence of albuminuria, is a significant complication of T2D marked by a sustained reduction in eGFR, increasing the risk of end-stage kidney disease (ESKD), which necessitates dialysis or kidney transplantation (3). Age-related changes cause a stable annual eGFR decline of 0.8–1.0 mL/min/1.73 m² in individuals over 40, while diabetic kidney disease (DKD) causes a more pronounced decline with median eGFR slopes ranging from 1.5 to 4.0 mL/min/1.73 m² annually (4). The rate of eGFR decline is a strong predictor of CKD progression and the likelihood of worse adverse kidney events (5–7). A prior study indicated that a rapid decline in eGFR, characterized by a reduction ≥5 mL/min/1.73 m² per year, was associated with higher risks of ESKD and mortality in both the general population and patients with diabetes (4, 8, 9).

Sodium glucose cotransporter 2 inhibitors (SGLT2is) have shown to be an effective treatment for patients with DKD (10–17). In addition to its glucose-lowering effects, SGLT2i exhibits reno-protective properties, such as slowing the rate of eGFR decline and lowering proteinuria, which reduces the risk of CKD progression and ESKD (10–13). Dipeptidyl peptidase-4 inhibitor (DPP4i) is a commonly T2D treatment that inhibits the dipeptidyl peptidase-4 enzyme, increasing insulin secretion and decreasing glucagon levels. Comparative real-world studies found that SGLT2is may be associated with a lower risk of ESKD and a slower decline in eGFR than DPP4is (18–24). However, the effect of SGLT2i vs. DPP4i on the risk of major composite adverse kidney outcomes in T2D patients with a prior rapid eGFR decline remains unclear. We hypothesized that SGLT2is would provide superior kidney protection than DPP4is in patients with T2D who had experienced varying rates of prior eGFR decline rates in real-world settings.

## Patients, materials, and methods

### Database

The current study used medical data from Chang Gung Memorial Hospital’s (CGMH) electronic database, Taiwan’s largest healthcare provider. The CGMH system, which includes two medical centers, two regional hospitals, and three district hospitals, has 10,050 beds and serves approximately 280,000 patients per year (25). The present study was approved by the Chang Gung Medical Foundation’s Institutional Review Board (202101936B0C503). The current study’s findings and interpretations do not reflect the position of the CGMH.

### Study design

**Figure 1:**
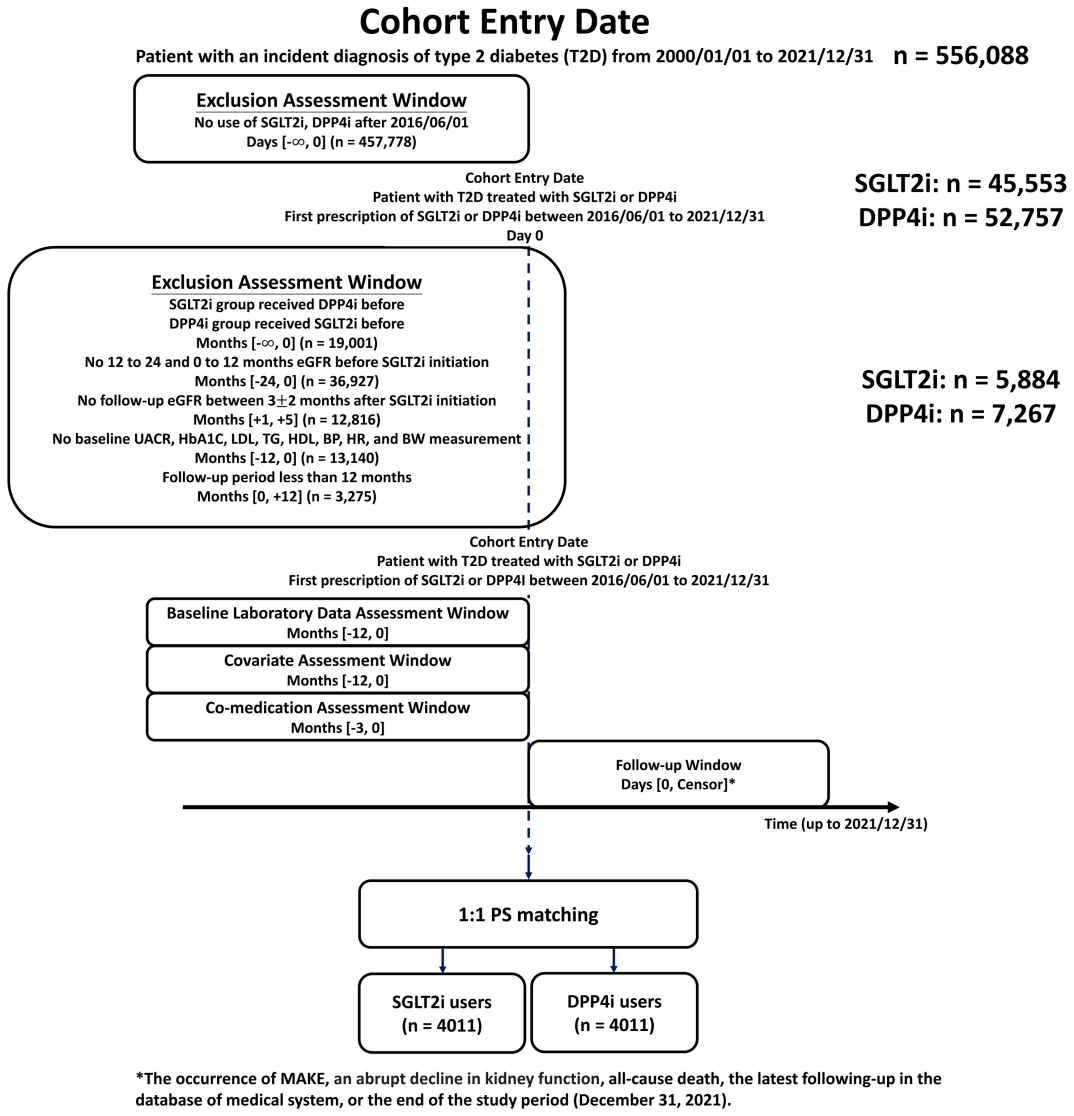
Study design and enrollment of people with type 2 diabetes (T2D) receiving sodium-glucose cotransporter 2 inhibitors (SGLT2i) and dipeptidyl peptidase-4 inhibitors (DPP4i). The study included 5,884 and 7,267 people with T2D undergoing SGLT2i and DPP4i therapy, respectively, who had at least one available eGFR measurement recorded at -24 to -12 and -12 to 0 months prior to the drug index date as well as 3-month follow-up eGFR measurements after the drug index date, from June 1, 2016 to December 31, 2021. There were 4,011 paired cohorts of SGLT2i versus DPP4i after propensity score matching (PSM). BP, blood pressure; BW, body weight; DPP4i, dipeptidyl peptidase-4 inhibitor; eGFR, estimated glomerular filtration rate; HbA1c, glycated hemoglobin; HDL, high-density lipoprotein; HR, heart rate; LDL, low-density lipoprotein; MAKE, major adverse renal event; PSM, propensity score matching; SGLT2i, sodium glucose cotransporter 2 inhibitor; T2D, type 2 diabetes; TG, triglyceride; UACR, urine albumin-to-creatinine ratio.

A total of 5,884 and 7,267 patients receiving SGLT2i and DPP4i treatment from 2016 to 2021 were eligible for the present study. **Figure 1** shows the flowchart summarizing the enrollment and study design. Between January 1, 2000 and December 31, 2021, we identified 556,088 people with an incident diagnosis of T2D. Because SGLT2i was approved later (May 1, 2016) than DPP4i in Taiwan, the drug index date was defined as the first prescription date for either of the two drugs after June 1, 2016 to ensure a timely comparison. During the same period, 45,553 and 52,757 patients received their first SGLT2i prescriptions (empagliflozin and dapagliflozin, approved on May 1, 2016, and canagliflozin, approved on March 1, 2018) and DPP4i prescriptions (sitagliptin, vildagliptin, saxagliptin, linagliptin, or alogliptin). Patients with T2D cannot use SGLT2i and DPP4i simultaneously or in combination due to financial constraints, according to the Taiwan’s National Health Insurance Regulations (26). Patients in the SGLT2i or DPP4i group who had previously been exposed to DPP4i and SGLT2i before the drug index date were excluded from the study. This study included patients receiving SGLT2i or DPP4i therapy who had at least one eGFR measurement available at -24 to -12 and -12 to 0 months prior to the drug index date as well as eGFR measurements taken 3 ± 2 months after the index date. We excluded patients who did not have baseline laboratory data available (-12 to 0 months prior to the drug index date), including urine albumin-to-creatinine ratio (UACR), serum hemoglobin A1c (HbA1c), lipid profile, blood pressure, resting heart rate, and body weight, all of which are associated with the risk of adverse kidney events. The study population was also restricted to patients with a minimum follow-up period of at least 12 months within the CGMH Medical System. Finally, the present study included 5,884 and 7,267 patients receiving SGLT2i and DPP4i, with baseline laboratory measures, past eGFR slope, and a minimum follow-up period of ≥12 months. The eGFR reported in this study was calculated with the CKD-EPI equation (2021) (27).

### Study outcomes

The subsequent clinical events occurred after the follow-up eGFR measurement was reported: (i) major adverse kidney event (MAKE), defined as a sustained 50% reduction in follow-up eGFR or the development of ESKD, defined as an eGFR of <15 ml/min^−1^/1.73 m^2^ during the follow-up period, and (ii) an abrupt decline in kidney function, defined as a pre-specified endpoint of doubling of serum creatinine between two subsequent eGFR measurements (28). The study only considered the study outcomes that occurred after the follow-up eGFR measurement date. The patients were followed up until the occurrence of study outcomes, mortality, the latest follow-up date documented in the CGMH medical system, or the end of the study period (December 31, 2021).

### Covariates

Any claims record containing the specified diagnoses or medication codes prior to the drug index date served as the baseline comorbidities. A prescription medication history was considered for drugs taken at least once in the 3 months preceding the drug index date. Baseline laboratory data, as shown in **Table 1**, were derived from measurements taken within 1 year of the drug index date. For those with multiple laboratory measurements within the year preceding the drug index date, the laboratory measurement nearest to the drug index date was adopted.

**Table 1:** Clinical characteristics of people with type 2 diabetes (T2D) treated with SGLT2i and DPP4i before and after propensity score matching (PSM).

	Before PSM			After PSM		
	SGLT2i (*n* = 5884)	DPP4i (*n* = 7267)	ASMD	SGLT2i (*n* = 4011)	DPP4i (*n* = 4011)	ASMD
Baseline characteristics						
Diabetes duration	8.4 ± 5.3	8.2 ± 5.6	0.036	8.3 ± 5.3	8.2 ± 5.6	0.016
Age (mean ± SD)	62.0 ± 11.6	67.9 ± 11.6	0.506	64.3 ± 10.7	64.6 ± 11.6	0.032
Male	3,515 (60)	3,859 (53)	0.134	2,252 (56)	2,253 (56)	0.001
Ischemic heart etiology	588 (10)	463 (6)	0.132	336 (8)	326 (8)	0.009
Cerebral vascular accidents	82 (1)	172 (2)	0.072	69 (2)	73 (2)	0.008
Congestive heart failure	255 (4)	224 (3)	0.066	139 (3)	136 (3)	0.004
Chronic lung disease	183 (3)	260 (4)	0.026	118 (3)	130 (3)	0.017
Chronic liver disease	1,996 (34)	2,254 (31)	0.062	1,328 (33)	1,331 (33)	0.002
Peripheral artery disease	60 (1)	77 (1)	0.004	38 (1)	40 (1)	0.005
Gout	822 (14)	992 (14)	0.009	550 (14)	564 (14)	0.010
Malignancy	547 (9)	1,119 (15)	0.186	420 (10)	457 (11)	0.030
Baseline vital signs						
Baseline height (cm)	162.1 ± 12.0	159.6 ± 12.7	0.203	161.3 ± 9.4	161.5 ± 9.3	0.019
Baseline body weight (KG)	74.8 ± 15.3	67.4 ± 13.0	0.520	71.5 ± 13.4	70.8 ± 13.7	0.052
Baseline SBP (mmHg)	139.7 ± 19.5	139.3 ± 20.2	0.016	139.6 ± 19.3	139.7 ± 19.9	0.003
Baseline DBP (mmHg)	78.2 ± 11.8	76.6 ± 12.5	0.131	77.5 ± 11.5	77.6 ± 12.3	0.010
Baseline heart rate (bpm)	84.0 ± 13.6	82.8 ± 13.6	0.087	83.3 ± 13.5	83.2 ± 13.5	0.010
Baseline laboratory data						
Pre-treatment eGFR slope (mL/min/1.73 m^2^/year) (med, IQR)	-1.33 (-4.45, 1.34)	-2.21 (-6.13, 0.24)	0.012	-1.48 (-4.72, 1.15)	-1.81 (-5.10, 0.50)	0.011
-24 to -12 month eGFR prior index date^a^ (mL/min/1.73 m^2^)	88.3 ± 21.0	79.6 ± 24.2	0.382	85.3 ± 20.9	84.8 ± 23.2	0.021
-12 to 0 month eGFR prior index date^a^ (mL/min/1.73 m^2^)	86.3 ± 21.1	75.6 ± 26.3	0.452	82.8 ± 21.0	82.2 ± 24.6	0.025
Baseline urine albumin-to-creatinine ratio (mg/g) (med, IQR)	23.7 (8.5, 112.0)	19.0 (7.8, 78.0)	0.452	21.8 (8.1, 102.2)	17.2 (7.4, 68.2)	0.025
Baseline HbA1c (%)	8.4 ± 1.6	7.6 ± 1.5	0.047	8.1 ± 1.4	8.0 ± 1.6	0.015
Baseline ALT (U/L)	35.8 ± 51.3	30.2 ± 26.3	0.492	33.9 ± 31.4	32.5 ± 27.8	0.058
Baseline triglycerides (mg/dL)	173.4 ± 199.8	152.3 ± 119.9	0.137	159.4 ± 112.4	156.9 ± 123.4	0.048
Baseline LDL (mg/dL)	91.6 ± 29.6	99.0 ± 70.9	0.128	92.6 ± 29.8	92.6 ± 37.0	0.021
Baseline HDL (mg/dL)	44.9 ± 11.7	46.4 ± 12.5	0.138	45.6 ± 11.8	45.8 ± 12.3	<.001
Baseline medications						
Use of anti-platelet agent	1,832 (31)	2,039 (28)	0.067	1,198 (30)	1,187 (30)	0.006
Use of statin	3,771 (64)	4,373 (60)	0.081	2,523 (63)	2,544 (63)	0.011
Use of CCB	1,024 (17)	1,579 (22)	0.109	743 (19)	760 (19)	0.011
Use of beta-blocker	2,080 (35)	2,077 (29)	0.146	1,297 (32)	1,279 (32)	0.010
Use of RAAS inhibitor	3,768 (64)	4,212 (58)	0.125	2,475 (62)	2,471 (62)	0.002
Use of loop diuretics	394 (7)	652 (9)	0.085	279 (7)	270 (7)	0.009
Use of thiazide	1,051 (18)	1,100 (15)	0.073	658 (16)	640 (16)	0.012
Use of MRA	202 (3)	197 (3)	0.042	121 (3)	112 (3)	0.013
Use of vasodilator	302 (5)	342 (5)	0.020	191 (5)	205 (5)	0.016
Use of NSAIDs	707 (12)	1,041 (14)	0.068	511 (13)	527 (13)	0.012
Use of UA lowering agent	553 (9)	819 (11)	0.062	396 (10)	430 (11)	0.028
Use of anti-diabetic agent						
Metformin	5,331 (91)	6,256 (86)	0.141	3,609 (90)	3,605 (90)	0.003
SU	3,117 (53)	3,032 (42)	0.227	1,964 (49)	1,936 (48)	0.014
Glinide	179 (3)	405 (6)	0.125	138 (3)	138 (3)	<.001
Glitazone	1,123 (19)	482 (7)	0.379	470 (12)	420 (10)	0.040
Acarbose	865 (15)	795 (11)	0.113	526 (13)	508 (13)	0.013
Insulin	1,001 (17)	846 (12)	0.154	559 (14)	538 (13)	0.015

Data are expressed as the mean ± standard deviation (SD), (med, IQR), or as percentage.

ASMD, absolute standardized mean difference; ALT, alanine aminotransferase; BMI, body mass index; CCB, calcium channel blocker; DBP, diastolic blood pressure; DPP4i, dipeptidyl peptidase-4 inhibitor; eGFR, estimated glomerular filtration rate; GLP-1RA, glucagon-like peptide 1 receptor agonist; HBA1c, hemoglobin A1c; HDL, high density lipoprotein; HR, heart rate; LDL, low density lipoprotein; MRA, mineralocorticoid receptor antagonist; NSAIDs, non-steroidal anti-inflammatory drugs; PSM, propensity score matching; RAAS, renin-angiotensin-aldosterone system; SBP, systolic blood pressure; SGLT2i, sodium glucose co-transporter-2 inhibitor; SU, sulfonylurea; T2D, type 2 diabetes, UA, uric acid; UACR, urine albumin-to-creatinine ratio.

a eGFR was calculated using the CKD-EPI 2021 equation.

### Statistical analysis

Continuous variables were presented as means and standard deviations (SD). Categorical variables were represented using proportions. ANOVA was used to analyze differences in continuous variables, while chi-square (*χ*^2^) test was used to compare nominal variables. To ensure a fair comparison between SGLT2i and DPP4i users, we used the propensity score matching (PSM) method, which pairs patients with similar characteristics in **Table 1** from each treatment group. This PSM method uses logistic regression to calculate each patient’s likelihood of receiving SGLT2i based on their baseline characteristics and then matches each SGLT2i user with the most similar DPP4i user (nearest-neighbor technique) in a 1:1 ratio without allowing any patient to be used more than once (29, 30). The PSM was used to rebalance the two study groups, resulting in matched sets with similar comorbidities, demographics, and medication profiles as listed in **Table 1**. This matching process creates two groups that are essentially comparable at baseline—like having two groups of patients who differ primarily in their medication choice rather than their underlying health status. This approach mimics some aspects of a randomized controlled trial by reducing confounding bias that could arise from systematic differences between patients who receive different treatments in clinical practice. Absolute standardized mean difference (ASMD) was used to compare potential confounding factors between matched study groups at the drug index date. This method was chosen over statistical testing because it focuses on assessing balance within the sample rather than drawing conclusions about the larger population. An ASMD value of ≤0.1 indicates no significant difference in potential confounders between the two paired study groups (31). The crude incidence rate was calculated by dividing the number of person-years at risk by the total number of observed study outcomes over the follow-up period. A Cox proportional hazards regression model was used to compare the risk of adverse events among patients who began SGLT2i or DPP4i treatment, stratified by their previous eGFR slope before treatment. The treatment effect of SGLT2i vs. DPP4i therapy on past eGFR slope was assessed using a linear regression model adjusted for baseline eGFR and UACR value and diabetes status. The efficacy of SGLT2i, compared to DPP4i therapy, on adverse kidney outcome was modeled as a fractional polynomial using the past eGFR slope as a continuous variable. The past eGFR slope before drug therapy and after 3 months of drug therapy was calculated using a linear regression model applied to all eGFR data from -24 months to the last available eGFR measurement before the drug index date and from 3 months to the last available eGFR measurement in each participant, respectively (9, 32). A two-sided *P*-value of <0.05 indicated statistical significance. Missing data of laboratory measurements at baseline (alanine aminotransferase (ALT)) will not be imputed for any of the baseline variables or study endpoints. All statistical analyses were carried out using SAS 9.4 (SAS Institute, Cary, NC, USA), SPSS 26.0 (IBM Corp., Armonk, NY, USA), or R Statistics 4.0.2 (R Foundation for Statistical Computing, Vienna, Austria).

## Results

### Baseline characteristics of patients receiving SGLT2i and DPP4i treatment

**Table 1** summarizes the baseline demographic characteristics of the two study groups before and after the PSM. Before PSM, there were significant differences in baseline characteristics between the two study groups (most ASMD >0.10). After PSM, there were 4,011 paired cohorts receiving SGLT2i and DPP4i treatment, respectively. The paired cohorts were well balanced in all baseline characteristics (all ASMD <0.10). **Supplementary Table S1** summarizes the baseline characteristics of the study population receiving SGLT2i and DPP4i therapy after PSM, categorized by past eGFR slope prior to drug therapy. During the follow-up period, SGLT2i therapy was associated with a reduction in blood pressure, resting heart rate, serum HbA1c, triglyceride, and low-density lipoprotein (LDL) as well as an increase in high-density lipoprotein (HDL) (*P* < 0.05), whereas DPP4i therapy was associated with a reduction in serum HbA1c, triglyceride, and LDL (*P* <.05). There were no changes in blood pressure, resting heart rate, and HDL following DPP4i therapy (**Supplementary Table S2**).

**Figure 2:**
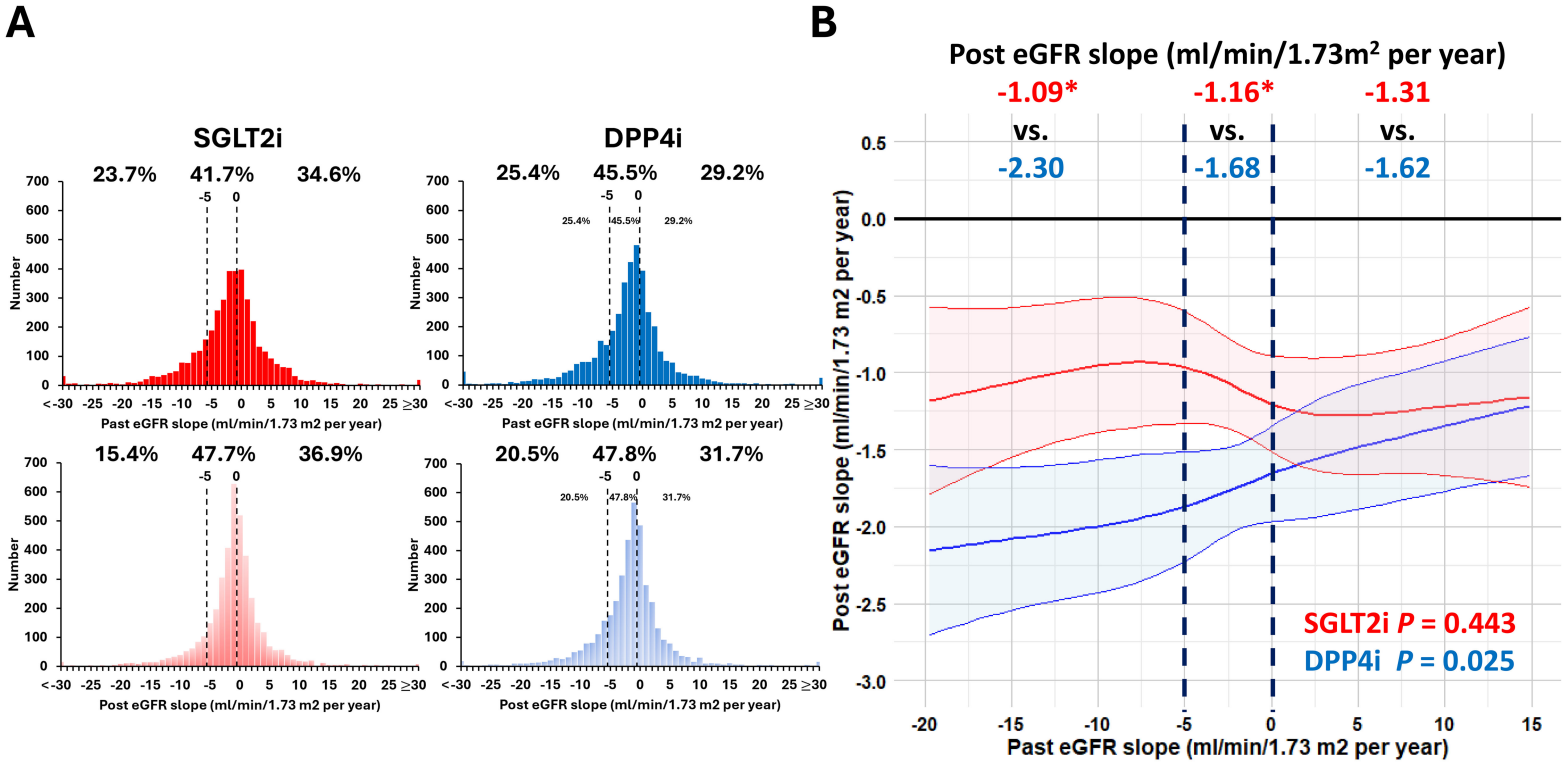
Changes of eGFR slope category before and after SGLT2i and DPP4i therapy **(A)** and post-treatment eGFR slope across the past eGFR slope in patients receiving SGLT2i and DPP4i therapy **(B)**. The study cohort was divided into three distinct groups based on their past eGFR slope: individuals with a past eGFR increase, past eGFR decrease 0 to 5, and past eGFR decrease ≥5 mL/min/1.73 m² per year. Following SGLT2i and DPP4i treatment, the proportion of patients experiencing rapid eGFR decline decreased from 23.7% (*n* = 951) to 15.4% (*n* = 616) and 25.4% (*n* = 1,017) to 20.5% (*n* = 824), respectively **(A)**. Patients receiving SGLT2i treatment were associated with a slower annual eGFR decline compared to those receiving DPP4i treatment (*P* < 0.001). In general, SGLT2i treatment was associated with a slower eGFR annual decline compared to DPP4i treatment, particularly in the subgroup with a past rapid eGFR decline prior to drug therapy. The shaded area represents the 95% confidence interval **(B)**. The abbreviations are as in **Figure 1**. *P < 0.05 vs. DPP4i treatment.

**Figure 2A** shows the proportion and detailed patient number of the study population, categorized by eGFR slope before and after starting SGLT2i or DPP4i therapy. The study cohort was divided into three distinct groups based on their past eGFR slope during the 2-year follow-up period: individuals with a past eGFR increase (34.6%, *n* = 1,388 for SGLT2i and 29.2%, *n* = 1,170 for DPP4i), past eGFR decrease 0 to 5 (41.7%, *n* = 1,672 for SGLT2i and 45.5%, *n* = 1,824 for DPP4i), and past eGFR decrease ≥5 (23.7%, *n* = 951 for SGLT2i and 25.4%, *n* = 1,017 for DPP4i) mL/min/1.73 m^2^ per year. The proportion of patients experiencing rapid eGFR decline decreased from 23.7% (*n* = 951) to 15.4% (*n* = 616) and 25.4% (*n* = 1,017) to 20.5% (*n* = 824), respectively, following the initiation of SGLT2i and DPP4i treatment (**Figure 2A**).

### Post-treatment eGFR slope according to different past eGFR slope in patients receiving SGLT2i and DPP4i treatment

SGLT2i treatment reduced the mean (SEM) eGFR decline from -1.84 (0.23) to -1.19 (0.10) mL/min/1.73 m^2^ per year (*P* = 0.011). Conversely, DPP4i treatment was not associated with a stabilization of the eGFR annual slope (from -2.09 (0.43) to -1.84 (0.23) mL/min/1.73 m^2^ per year (*P* = 0.55)). Patients receiving SGLT2i treatment were associated with a slower annual eGFR decline compared to those receiving DPP4i treatment (*P* < 0.001). In general, SGLT2i treatment was associated with a slower eGFR annual decline compared to DPP4i treatment across the range of past eGFR slope examined as a continuous variable, especially in conditions with rapid eGFR decline of ≥5 mL/min/1.73 m^2^ per year. Both SGLT2i and DPP4i treatment improved the eGFR decline in patients with past rapid eGFR decline from -11.41 (0.40) to -1.09 (0.24) mL/min/1.73 m^2^ per year (*P* < 0.001) and -12.06 (0.42) to -2.30 (0.25) mL/min/1.73 m^2^ per year (*P* < 0.001), respectively (**Figure 2B**).

### Risk of adverse kidney outcomes across different categories of past eGFR decline in patients receiving SGLT2i vs. DPP4i treatment

**Figure 3:**
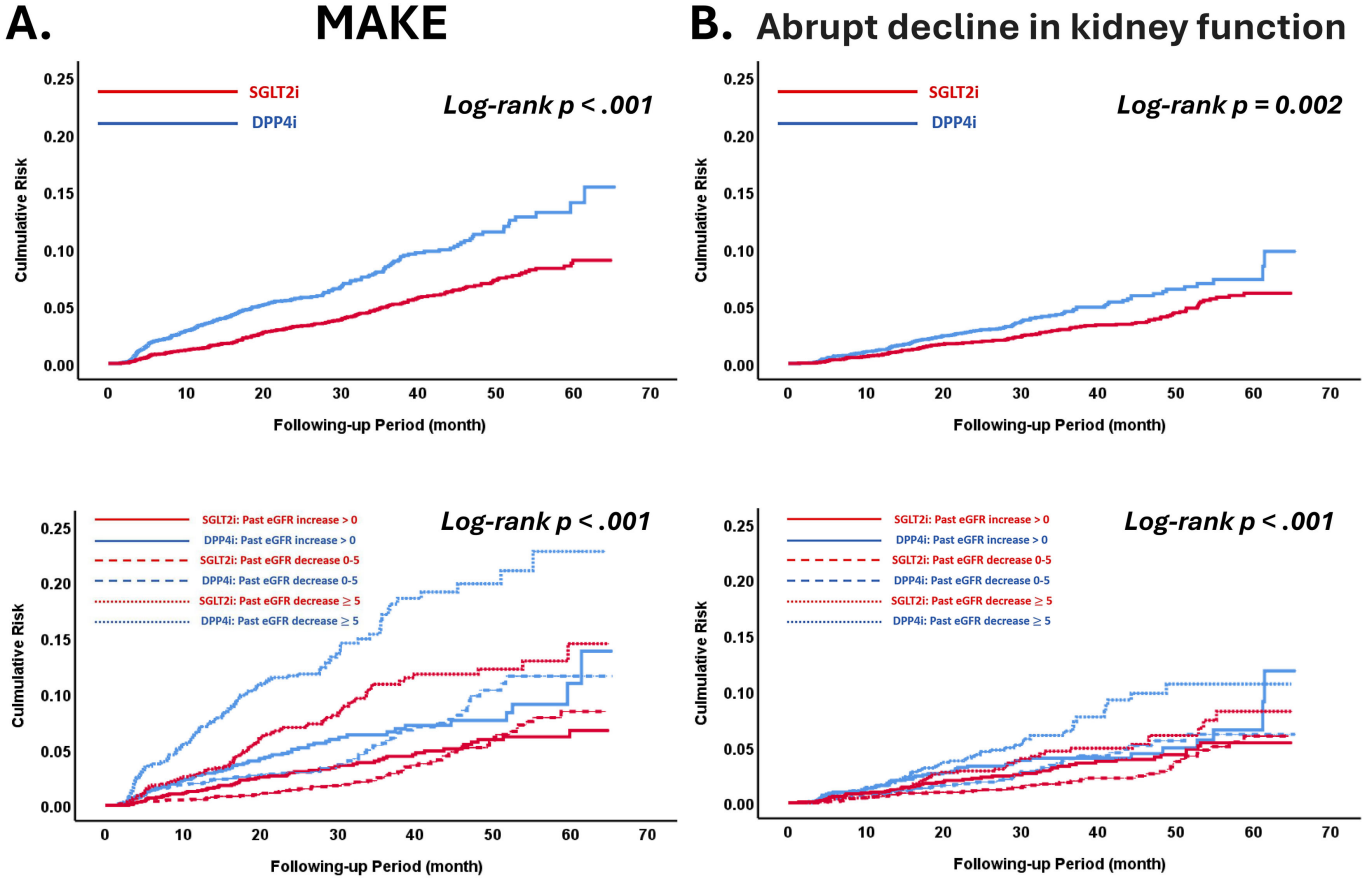
The cumulative risk of a major adverse kidney event **(A)** and an abrupt decline in kidney function **(B)** for the paired study cohorts receiving SGTL2i vs. DPP4i after PSM SGLT2i treatment was associated with lower risks of incident MAKE **(A)** and an abrupt decline in kidney function **(B)** compared with DPP4i treatment in people with T2D after PSM, consistent in subgroups with varying past eGFR slopes before drug therapy. The abbreviations are as in **Figure 1**.

**Figure 4:**
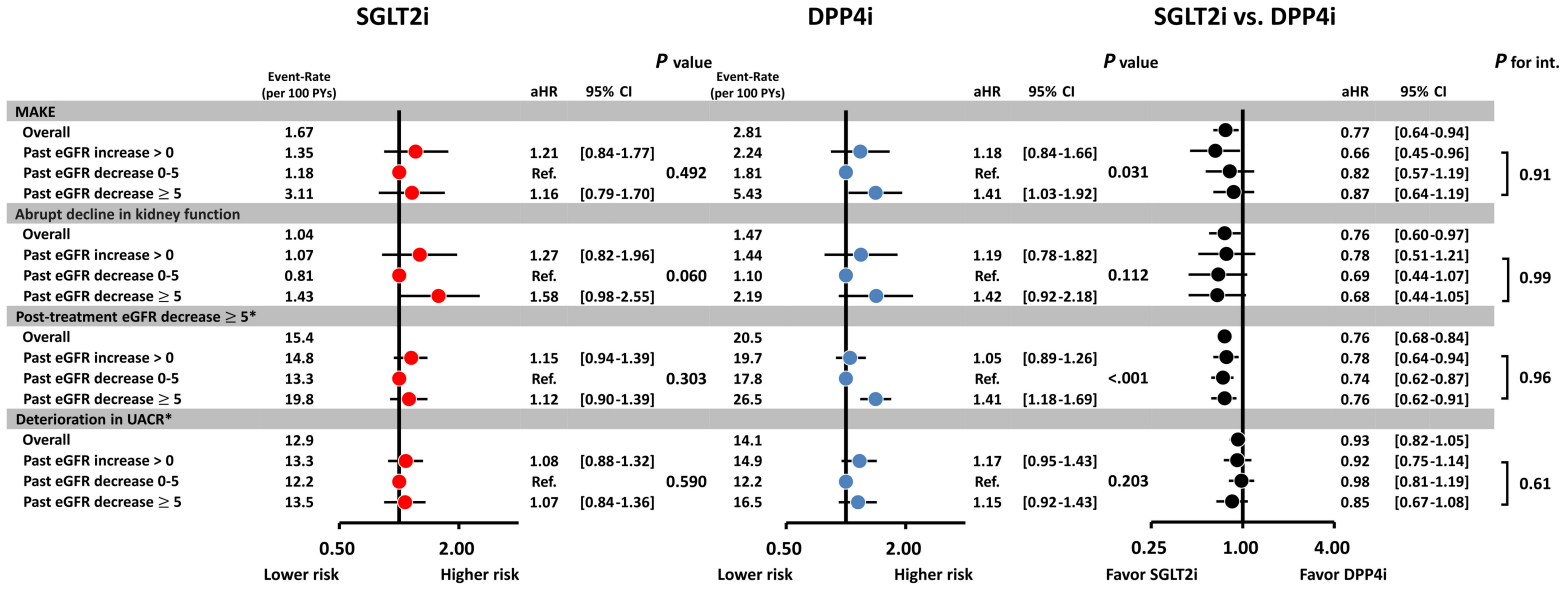
Risk of adverse kidney outcomes for the paired study cohorts receiving SGLT2i vs. DPP4i after PSM according to different categories of past eGFR slope prior to drug treatment. Overall, the participants treated with SGLT2i were associated with a lower risk of adverse kidney outcomes compared with those treated with DPP4i, and the treatment benefit was persistent across three categories of past eGFR slope before drug treatment. There was no difference in the risk of deterioration of UACR for SGLT2i vs. DPP4i treatment. aHR, adjusted hazard ratio; CI, confidence interval. Other abbreviations are as in **Figure 1**. *, post-treatment eGFR decrease ≥5 mL/min/1.73 m² per year and deterioration in UACR are expressed as event-rate per 100 patients. #, risk of outcomes was adjusted for age, gender, duration of diabetes, all baseline comorbidities, baseline body weight, HbA1c, eGFR, UACR, lipid profile, systolic blood pressure, heart rate, and all baseline cardiovascular drugs and anti-hyperglycemic agents in **Table 1**.

Patients receiving SGLT2i treatment were associated with a lower cumulative risk of developing MAKE and an abrupt decline in kidney function than those receiving DPP4i treatment, and the treatment benefit was consistent across three categories of past eGFR slope before drug treatment (**Figure 3**). Patients with a rapid past eGFR decline of ≥5 mL/min/1.73 m² per year were associated with a higher risk of MAKE and post-treatment eGFR decline of ≥5 mL/min/1.73 m² per year compared to those with a past eGFR slope decline of 0 to 5 mL/min/1.73 m² per year. However, this was not observed in patients receiving SGLT2i treatment. Overall, the participants treated with SGLT2i were associated with a lower risk of MAKE (hazard ratio [HR], 0.77; 95% confidence interval [CI], 0.64 to 0.94), an abrupt decline in kidney function (HR, 0.76; 95% CI, 0.60 to 0.97), and post-treatment eGFR decline of ≥5 mL/min/1.73 m^2^ per year (HR, 0.76; 95% CI, 0.68 to 0.84) compared with those treated with DPP4i, and the treatment benefit was persistent across three categories of past eGFR slope before drug treatment (*P* for interaction—all >0.05). There was no difference in the risk of UACR deterioration between SGLT2i and DPP4i treatment across the different categories of pre-treatment eGFR slope (**Figure 4**). There was no difference in the risk of all kidney outcomes between empagliflozin and dapagliflozin treatment across the different categories of pre-treatment eGFR slope (**Supplementary Table S3**). We have performed sensitivity analysis considering adjusting alanine aminotransferase (ALT) at baseline as the covariate factor, showing that the treatment benefit was consistent across three categories of past eGFR slope before drug treatment (*P* for interaction all >.05) (**Supplementary Table S4**). We have also performed further sensitivity analyses considering different SGLT2i drugs (empagliflozin or dapagliflozin) or different definition of MAKE, indicating that the treatment benefit for SGLT2i vs. DPP4i was consistent across three categories of past eGFR slope before drug treatment (*P* for interaction, all >.05) (**Supplementary Tables S5–S7**).

### Non-linear relationship in risk of adverse kidney outcomes with different past eGFR decline in patients receiving SGLT2i vs. DPP4i treatment

**Figure 5:**
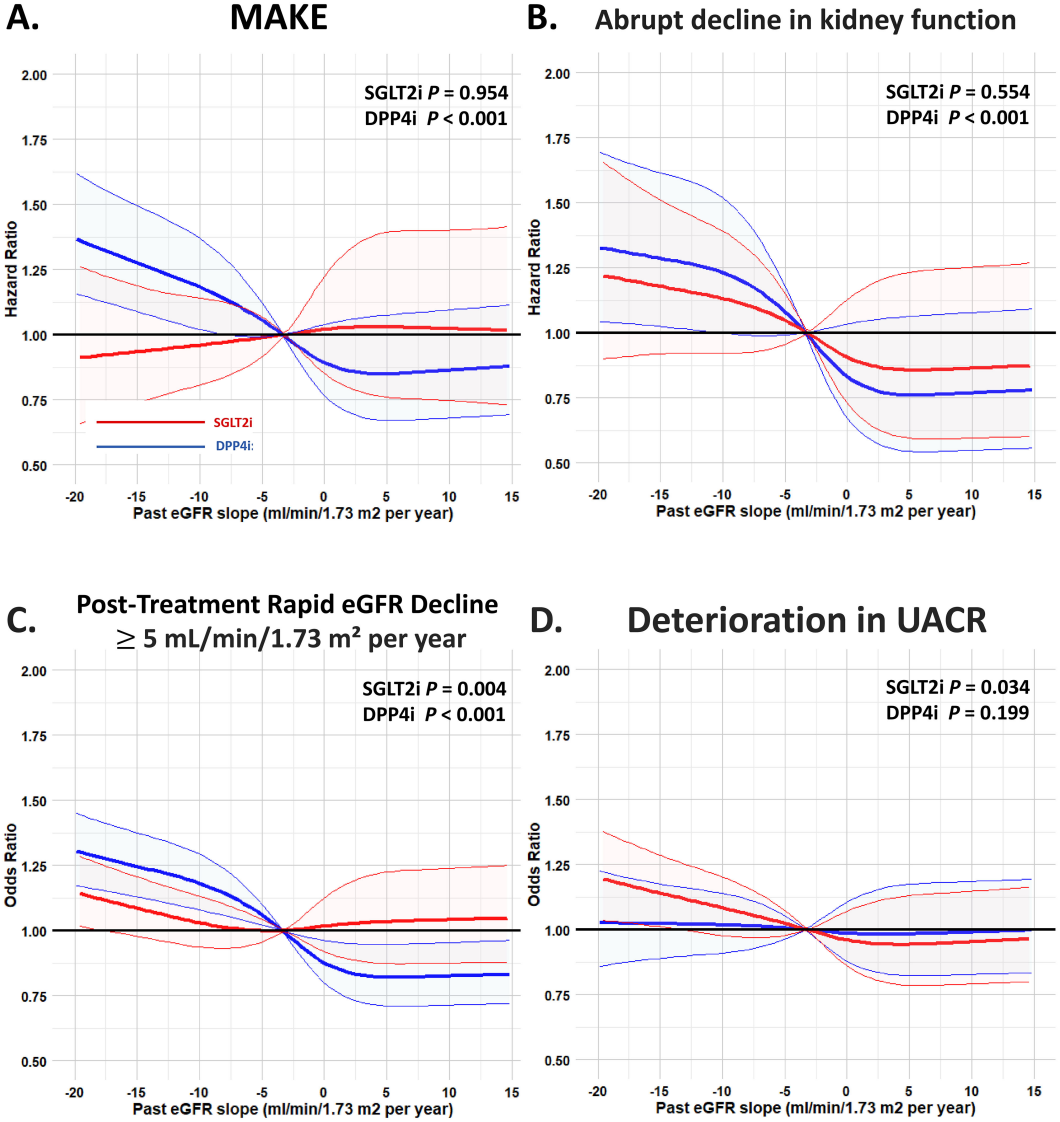
Risk of adverse kidney outcomes for the paired study cohorts receiving SGLT2i vs. DPP4i after PSM across the range of different past eGFR slope examined as a continuous variable. Modeling the different past eGFR slope before drug initiation as a continuous variable with a restricted cubic splines model; a more obvious eGFR decline slope before index-drug initiation was independently associated with a higher risk of MAKE **(A)** and an abrupt decline in kidney function **(B)** in patients receiving DPP4i therapy (*P* both <0.05) but not in those receiving SGLT2i therapy. The abbreviations are as in **Figure 1**. #, adjusted factor as in **Figure 4**. *, post-treatment eGFR decrease ≥5 mL/min/1.73 m² per year and deterioration in UACR are expressed as event-rate per 100 patients. **(C)** Post-Treatment Rapid eGFR Decline ≥5 mL/min/1.73 m² per year. A more obvious eGFR decline slope before index-drug initiation was independently associated with a higher risk of post-treatment rapid eGFR decline ≥ 5 mL/min/1.73 m^2^ per year in patients receiving SGLT2i and DPP4i therapy. **(D)** Deterioration in UACR. A more obviouse GFR decline slope before index-drug initiation was independently associated with a higher risk of deterioration in UACR in patients receiving SGLT2i therapy but not in those receiving DPP4i therapy.

**Figure 6:**
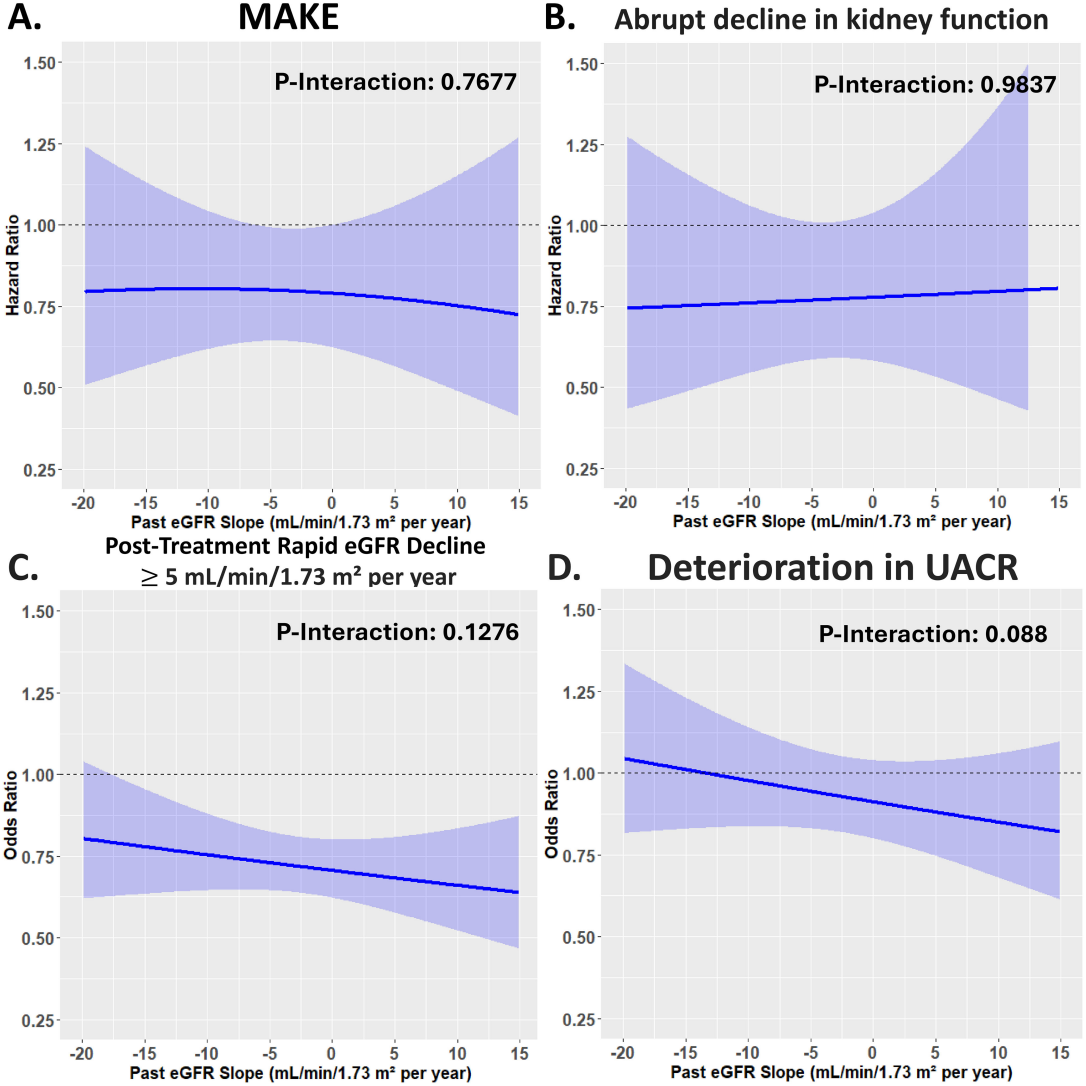
Risk of adverse kidney outcomes for the paired study cohorts receiving SGLT2i vs. DPP4i after PSM across the range of past eGFR slope examined as a continuous variable. The benefit of SGLT2i compared with DPP4i therapy was consistent for several kidney outcomes across the range of past eGFR slope examined as a continuous variable. The abbreviations are as in **Figure 1**. #, the adjusted factor is as in **Figure 4**. *, post-treatment eGFR decrease ≥5 mL/min/1.73 m² per year and deterioration in UACR are expressed as event-rate per 100 patients. **(A)** MAKE. The benefit of SGLT2i compared with DPP4i therapy was consistent across the range of past eGFR slope examined as a continuous variable. **(B)** Abrupt decline in kidney function. The benefit of SGLT2i compared with DPP4i therapy was consistent across the range of past eGFR slope examined as a continuous variable. **(C)** Post-Treatment Rapid eGFR Decline ≥5 mL/min/1.73 m² per year. The benefit of SGLT2i compared with DPP4i therapy was consistent across the range of past eGFR slope examined as a continuous variable. **(D)** Deterioration in UACR. The benefit of SGLT2i compared with DPP4i therapy was consistent across the range of past eGFR slope examined as a continuous variable.

Modeling the different past eGFR slope before drug initiation as a continuous variable with a restricted cubic splines model, we observed that patients receiving DPP4i treatment was associated with a higher risk of MAKE and an abrupt decline in kidney function compared to those receiving SGLT2i treatment (*P* both <0.001). In general, the upward trend in risks of adverse kidney outcomes with a decrement in past eGFR slope prior to drug initiation was steeper for DPP4i patients than for SGLT2i patients (**Figure 5**). The benefit of SGLT2i compared with DPP4i therapy was consistent for several kidney outcomes including MAKE, an abrupt decline in kidney function, and a post-treatment eGFR decline of ≥5 mL/min/1.73 m^2^ per year across the range of past eGFR slope examined as a continuous variable (**Figure 6**).

## Discussion

In the present study, we analyzed 4,011 matched individuals with T2D receiving SGLT2i and DPP4i treatments to assess changes in the eGFR slope before and after treatment as well as several adverse kidney outcomes. Overall, SGLT2i treatment was consistently associated with a slower annual decline in eGFR than DPP4i treatment, regardless of past eGFR slopes before drug therapy. SGLT2i and DPP4i therapy reduced the risk of rapid eGFR decline (≥5 mL/min/1.73 m² per year) after starting the treatment. Compared to patients with a past eGFR slope decline of 0–5 mL/min/1.73 m² per year, those with a rapid past eGFR decline were associated with worse adverse kidney outcomes among patients receiving DPP4i treatment, but not SGLT2i treatment. Overall, the participants treated with SGLT2i were associated with a lower risk of MAKE, abrupt kidney function decline, and persistent post-treatment rapid eGFR decline than those treated with DPP4i, with these benefits consistent across different past eGFR slope categories. The treatment benefits of SGLT2i over DPP4i were consistent across a range of past eGFR slopes examined as a continuous variable.

A rapid decline in eGFR has a significant impact on clinical outcomes, increasing the risk of kidney disease progression and cardiovascular complications (5, 33). While most patients with T2D experience gradual kidney function decline over time, some experience it more quickly, increasing the risk of kidney failure and cardiovascular events. Rapid eGFR decline increases the risk of mortality as well as macrovascular and microvascular complications (34–36). The mechanisms underlying rapid eGFR decline in patients with T2D are complex and may vary by patient, including hypertension, high urinary albumin levels, poor glycemic control, or specific glomerular pathological features (34, 37, 38). Furthermore, high baseline eGFR, older age, female sex, smoking, and retinopathy have been identified as predictors of rapid eGFR decline in patients with T2D (37).

Managing rapid eGFR decline in patients with T2D focuses on addressing underlying causes and slowing the rate of kidney function decline, which includes optimizing blood pressure, strict glycemic control, weight loss, and the use of kidney-protective medications such as RAAS inhibitor (39–41). The timing of SGLT2i initiation relative to the eGFR decline status appears to influence both the magnitude and mechanisms of treatment response in patients with T2D. When SGLT2i was initiated early before significant loss of kidney function (e.g., eGFR ≥ 60 mL/min/1.73 m²), SGLT2is provides robust glucose-lowering effects through increased glucosuria while concurrently activating renoprotective pathways that reduce intraglomerular pressure, inflammation, and oxidative stress. Early initiation therefore not only improves metabolic control but may also slow the onset of albuminuria and delay DKD progression. In contrast, when SGLT2i was introduced later in the disease course (e.g., eGFR < 30 mL/min/1.73 m²), the glycemic efficacy diminishes because of reduced filtered glucose load; however, substantial renal and cardiovascular protection persists, driven by hemodynamic and anti-fibrotic mechanisms independent of glucose lowering. Previous clinical trials consistently demonstrate benefit across a wide eGFR range, even at low kidney function levels (10–17). Nevertheless, initiating SGLT2i therapy earlier in the course of eGFR decline is likely to maximize long-term preservation of kidney function and reduce adverse cardiorenal outcomes, emphasizing the importance of timely therapy to optimize both metabolic and organ-protective effects. Despite large pivotal trials demonstrating that SGLT2i therapy is highly effective in slowing chronic eGFR decline and improving composite kidney outcomes in patients with T2D or CKD (10–17), it is unclear whether SGLT2i therapy can reverse rapid eGFR decline in a T2D patient at risk. In a *post-hoc* analysis of the EMPA-REG OUTCOME trial, Hadjadj S et al. investigated the impact of empagliflozin on the incidence of a “rapid decliner” phenotype (annual eGFR decline greater than 3 mL/min/1.73 m²). The results showed that empagliflozin treatment was associated with a lower incidence of this phenotype compared to placebo, with 3.4% of patients taking empagliflozin experiencing rapid decline versus 9.5% in the placebo group. A similar risk reduction was observed when using a more stringent threshold of rapid eGFR decline of 5 mL/min/1.73 m², consistent with the findings in our study (42).

Another small real-world study investigated the kidney-protective effects of SGLT2is in 165 patients with T2D who had moderate or rapid eGFR decline but retained kidney function prior to drug treatment. Among 165 patients, 21 had a history of an annual eGFR decline of ≥5 mL/min/1.73 m² before starting SGLT2i therapy, defining them as rapid decliners. Rapid decliners on SGLT2i have significantly improved their average annual eGFR slope compared to the control group (-4.36 vs. -1.00 mL/min/1.73 m² per year; *P* < 0.001). Notably, the steeper the eGFR slope before starting SGLT2i therapy, the greater the improvement in the eGFR slope, regardless of the albuminuria reduction (43). Another study examined the kidney-protective effects of SGLT2is in 85 patients with T2D and CKD at baseline, with varying rates of past eGFR decline prior to SGLT2i treatment. The study examined changes in eGFR slopes over 2 years before and during the 2-year treatment period and found that the rate of annual eGFR decline slowed significantly after starting SGLT2i therapy compared to before treatment. This kidney protection was independent of the patients’ demographic characteristics, albuminuria levels, and baseline eGFR. Despite varying past eGFR slopes prior to SGLT2i therapy, post-treatment eGFR slopes were similar across the three groups at 3 months later. Even for patients with rapid eGFR decline (≥3 mL/min/1.73 m² per year) before starting SGLT2i therapy, the drug significantly reduced the rate of eGFR decline (44).

Several studies compared SGLT2i to DPP4i in terms of chronic eGFR changes and kidney outcomes in patients with T2D (18–24). These studies consistently show that SGLT2i therapy improves kidney outcomes more than DPP4i therapy does. While *in vitro* and animal studies show that DPP4i may possess anti-inflammatory and antifibrotic properties in the kidney (45), these kidney-protection effects have not been consistently confirmed in human studies. Several clinical trials found that DPP4i therapy may have a positive impact on kidney outcomes, including an improvement and/or less deterioration in UACR categories, regardless of its effect on glycemic control (46–48). Nonetheless, there were no significant differences in eGFR changes or the occurrence of safety kidney endpoints in those studies (46–48). The abovementioned findings are consistent with our findings, which showed that SGLT2i treatment reduced the risk of major composite kidney outcomes when compared to DPP4i treatment, but there was no significant difference in the risk of UACR deterioration between the two groups, indicating that SGLT2i’s renal protection mechanisms may encompass pathways other than the recognized UACR reduction effects.

Our study showed that SGLT2i therapy was associated with a lower risk of major adverse kidney events and abrupt decline in kidney function when compared with DPP4i therapy in patients with T2D, while no significant difference was observed between groups for UACR progression. This discrepancy may be explained by the different pathways through which SGLT2is exerts kidney protection (49–51). The treatment benefits of SGLT2 inhibition are primarily mediated by hemodynamic and tubular mechanisms, such as reduction of intraglomerular pressure, improved tubuloglomerular feedback, and attenuation of renal hypoxia and fibrosis, which translate into preservation of long-term eGFR. These mechanisms may not consistently lead to reductions in albuminuria, which is a more variable biomarker influenced by glycemic control, blood pressure, dietary factors, and intercurrent illness. In addition, comparator effects may have contributed: although DPP4 inhibitors are generally considered neutral with respect to hard kidney outcomes, several studies have suggested modest reductions in albuminuria for DPP4i treatment (46–48). If DPP4i therapy already lowers UACR modestly, the gap in albuminuria progression between treatment groups may narrow, even while clinically meaningful differences in harder kidney outcomes remain evident. Nevertheless, our conclusion highlights that the UACR progression does not fully capture the renoprotective benefits of SGLT2 inhibition, emphasizing the importance of evaluating both albuminuria and hard kidney outcomes in patients with T2D receiving drug therapy.

Despite the fact that the abovementioned studies demonstrate the treatment benefit of several kidney outcomes associated with SGLT2i therapy over DPP4i therapy, no study that specifically focused on patients with varying past eGFR decline slope was available. Only one study investigated the effects of subgroups with varying pre-index eGFR change on kidney outcomes (18). Patients with (23.9% for study group) or without rapid decline in eGFR (≥5 mL/min/1.73m^2^ per year) were associated with lower risks of ESKD, acute kidney failure, and a comparable risk of albuminuria progression for SGLT2i vs. DPP4i therapy, consistent with the findings in our study. Further study is warranted to determine whether rapid eGFR decliners would benefit more from SGLT2i therapy.

### Study limitations

The present study has several limitations. The study was conducted on Asians exclusively, which may limit the generalizability of the findings to non-Asians, who may have different genetic predispositions and environmental exposures on kidney outcomes. The present study was conducted within a single healthcare system, which may potentially restrict generalizability to other populations and healthcare settings. Due to its retrospective and observational design, the present study is susceptible to the inherent biases linked to the hospital-based database analyses. Participants classified into distinct past eGFR change groups receiving SGLT2i or DPP4i therapy exhibited varied baseline clinical characteristics, and although adjustments were made for baseline characteristics, vital signs, laboratory data, and comedications through the PSM model, the potential for residual or unmeasured confounding factors remains. In addition, the CGMH database lacks lifestyle data. Unmeasured lifestyle factors such as sodium intake, high protein diet, smoking, physical inactivity, and medication nonadherence can have an adverse impact on short-term and long-term kidney outcomes in people with diabetes, thereby accelerating the progression to DKD and ESKD (39). Furthermore, baseline ALT measurements were available for only 7,816 patients (97.4%) of the total 8,022 study participants, with 206 patients (2.6%) having missing ALT data. Complete data was available for all other laboratory variables presented in **Table 1** across all 8,022 patients. Baseline ALT was excluded from the Cox regression model, which may represent a limitation since liver-related factors such as hepatorenal syndrome and drug-induced hepatotoxicity can influence kidney function. Regarding the measurement limitations, kidney function was assessed using serial serum creatinine-based eGFR slopes rather than cystatin C, which may be influenced by fluctuations in muscle mass or body composition. We did not consider the potential impact of temporal variations in follow-up laboratory data, medical diagnoses, or medications on the clinical outcomes observed during the study period since those factors may mediate kidney outcomes. Our current study did not compare five different DPP4is (sitagliptin, vildagliptin, saxagliptin, linagliptin, and alogliptin). Stratifying by individual DPP4is would result in extremely small sample sizes for each DPP4i, significantly increasing the risk of bias and type II error. Given the shared mechanism of action and similar efficacy profiles of DPP4i demonstrated in previous clinical studies, we investigated DPP4 inhibitors as a therapeutic class for the study design. Finally, the number of patients using GLP-1 receptor agonists was very limited in our cohort, precluding meaningful comparative analyses with this drug class; future studies with larger sample sizes across diverse antidiabetic medications are warranted.

## Conclusions

In this real-world study of people with T2D, SGLT2i therapy was associated with lower risks of MAKE, abrupt kidney function decline, and persistent post-treatment rapid eGFR decline. These benefits were consistent regardless of whether patients had experienced rapid eGFR decline prior to treatment.

## Data Availability

The original contributions presented in the study are included in the article/**Supplementary Material**, further inquiries can be directed to the corresponding author/s.
