# Supplementary material for: Effect of SGLT2 inhibitors versus DPP4 inhibitors on major adverse kidney events in diabetic people with varied kidney function decline
Source: Front Endocrinol (Lausanne). 2026 Jan 15;16:1647342. doi: 10.3389/fendo.2025.1647342 (PMC12852007; doi:10.3389/fendo.2025.1647342)
Supplement: Supplementary file 1 [file DataSheet1.pdf]

Supplemental Table S1 Clinical characteristics of the people with type 2 diabetes (T2D) treated with SGLT2i and DPP4i after propensity score matching (PSM), categorized by past eGFR slope prior to drug therapy.

	SGLT2i (n = 4011)			DPP4i (n = 4011)		
	No past-eGFR Decline (n = 1388)	Past eGFR 0 to 5 ml/min/1.73 m^2/year Decline (n = 1672)	Past eGFR ≥ 5 ml/min/1.73 m^2/year Decline (n = 951)	No past-eGFR Decline (n = 1170)	Past eGFR 0 to 5 ml/min/1.73 m^2/year Decline (n = 1824)	Past eGFR ≥ 5 ml/min/1.73 m^2/year Decline (n = 1017)
**Baseline characteristics**						
**Diabetes duration**	8.2±5.3	8.2±5.6	8.3±5.4	7.8±5.6	8.4±5.6	8.4±5.6
**Age (mean ± SD)**	64.9±10.9	63.9±10.3	64.0±11.1	65.0±11.8	64.1±11.4	65.2±11.8
**Male**	790 (57)	920 (53)	542 (50)	670 (57)	972 (53)	611 (60)
**Ischemic heart etiology**	117 (8)	116 (6)	103 (11)	92 (8)	115 (6)	119 (12)
**Cerebral vascular accidents**	20 (1)	21 (2)	28 (3)	23 (2)	29 (2)	21 (2)
**Congestive heart failure**	44 (3)	47 (3)	48 (5)	37 (3)	36 (2)	63 (6)
**Chronic lung disease**	51 (4)	40 (4)	27 (3)	48 (4)	42 (2)	40 (4)
**Chronic liver disease**	486 (35)	562 (31)	280 (29)	412 (35)	588 (32)	331 (33)
**Peripheral artery disease**	16 (1)	14 (1)	8 (1)	14 (1)	10 (1)	16 (2)
**Gout**	197 (14)	213 (14)	140 (15)	162 (14)	234 (13)	168 (17)
**Malignancy**	157 (11)	169 (15)	94 (10)	132 (11)	194 (11)	131 (13)
**Baseline vital signs**						
**Baseline height (cm)**	162.2±10.3	161.4±8.8	161.3±9.1	161.6±9.4	161.1±9.0	161.9±9.5
**Baseline body weight (KG)**	71.1±13.6	71.5±13.5	71.9±12.8	70.9±14.1	70.2±13.2	71.6±14.1
**Baseline SBP (mmHg)**	139.3±18.9	139.7±19.1	140.0±20.4	139.3±19.8	139.4±19.0	140.6±21.3
**Baseline DBP (mmHg)**	77.4±12.1	77.5±10.8	77.6±11.9	77.6±12.3	77.6±11.9	77.6±12.9

**Baseline heart rate (bpm)**	82.6±13.5	84.0±13.3	83.2±13.9	82.9±13.4	83.2±13.4	83.5±13.8
**Baseline laboratory data**						
**Pre-treatment eGFR slope (ml/min/1.73 m^2 per year) (med, IQR)**	2.56 (1.03, 5.75)	-2.00 (-3.32, -1.00)	-8.52 (-12.39, -6.49)	2.30 (1.04, 5.24)	-1.99 (-3.13, -1.01)	-8.97 (-12.86, -6.71)
**-24 to -12 month eGFR prior index date* (ml/min/1.73 m^2)**	83.2±20.8	89.2±20.4	81.5±20.9	84.2±22.1	89.0±22.3	78.2±24.1
**-12 to 0 month eGFR prior index date* (ml/min/1.73 m^2)**	85.7±19.4	86.7±20.6	71.7±20.1	87.4±21.2	86.9±22.9	67.8±25.6
**Baseline UACR (mg/g) (med, IQR)**	19.8 (8.0, 79.1)	19.55 (7.5, 79.0)	37.7 (10.5, 243.6)	16.6 (7.5, 49.5)	13.8 (6.8, 48.8)	28.0 (9.2, 180.0)
**Baseline HbA1c (%)**	8.1±1.3	8.1±1.3	8.0±1.4	8.1±1.7	7.9±1.6	7.9±1.6
**Baseline ALT (U/L)**	34.9±35.4	33.3±25.1	33.4±35.0	34.0±31.0	32.5±27.2	30.7±24.5
**Baseline Triglycerides (mg/dL)**	153.9±105.7	153.5±107.7	177.7±127.1	159.0±123.1	148.2±115.2	170.0±136.2
**Baseline LDL (mg/dL)**	90.9±30.1	93.2±28.5	94.0±31.6	92.8±37.7	93.4±37.8	90.9±34.7
**Baseline HDL (mg/dL)**	45.6±11.8	46.2±11.6	44.8±12.0	45.1±12.1	47.0±12.2	44.5±12.5
**Baseline medications**						
**Use of anti-platelet agent**	445 (32)	435 (26)	318 (33)	357 (31)	474 (26)	356 (35)
**Use of statin**	867 (62)	1085 (65)	571 (60)	735 (63)	1159 (64)	650 (64)
**Use of CCB**	242 (17)	295 (18)	206 (22)	232 (20)	307 (17)	221 (22)
**Use of beta-blocker**	461 (33)	466 (28)	370 (39)	364 (31)	510 (28)	405 (40)
**Use of RAAS inhibitor**	860 (62)	963 (58)	652 (69)	697 (60)	1067 (58)	706 (69)
**Use of loop diuretics**	89 (6)	79 (5)	111 (12)	70 (6)	79 (4)	121 (12)
**Use of thiazide**	196 (14)	270 (16)	192 (20)	183 (16)	242 (13)	215 (21)
**Use of MRA**	36 (3)	37 (2)	48 (5)	33 (3)	29 (2)	50 (5)
**Use of vasodilator**	64 (5)	64 (4)	63 (7)	47 (4)	72 (4)	86 (8)
**Use of NSAIDs**	182 (13)	189 (11)	140 (15)	156 (13)	226 (12)	145 (14)

**Use of uric-acid lowering agent**	142 (10)	139 (8)	115 (12)	124 (11)	172 (9)	134 (13)
**Use of anti-diabetic agent**						
**Metformin**	5331 (90)	6256 (91)	841 (88)	1055 (90)	1670 (92)	880 (87)
**SU**	3117 (46)	3032 (51)	462 (49)	561 (48)	863 (47)	512 (50)
**Glinide**	179 (3)	405 (3)	40 (4)	45 (4)	39 (2)	54 (5)
**Glitazone**	1123 (12)	482 (11)	126 (13)	118 (10)	180 (10)	122 (12)
**Acarbose**	865 (13)	795 (13)	132 (14)	163 (14)	215 (12)	130 (13)
**Insulin**	1001 (13)	846 (13)	153 (16)	168 (14)	191 (10)	179 (18)

ASMD, absolute standardized mean difference; ALT, alanine aminotransferase; BMI, body mass index; CCB, calcium channel blocker; DBP, diastolic blood pressure; DPP4i, dipeptidyl peptidase-4 inhibitor; eGFR, estimated glomerular filtration rate; GLP-1RA, glucagon-like peptide 1 receptor agonist; HBA1c, hemoglobin A1c; HDL, high density lipoprotein; HR, heart rate; LDL, low density lipoprotein; MRA, mineralocorticoid receptor antagonist; NSAIDs, non-steroidal anti-inflammatory drugs; PSM, propensity score matching; RAAS, renin-angiotensin-aldosterone system; SBP, systolic blood pressure; SGLT2i, sodium glucose co-transporter-2 inhibitor; SU, sulfonylurea; T2D, type 2 diabetes, UACR, urine albumin-to-creatinine ratio

Data are expressed as the mean ± standard deviation (SD), (med, IQR), or as percentage.

*The eGFR was calculated using the CKD-EPI 2021 equation

Supplemental Table S2 Baseline and endpoint laboratory values people with type 2 diabetes following SGLT2i and DPP4i treatment

	SGLT2i (n = 4011)			DPP4i (n = 4011)		
	**Baseline**	**End**	***P* value**	**Baseline**	**End**	***P* value**
**SBP (mmHg)**	139.6±19.3	136.5±19.2	<.001	139.7±19.9	139.0±19.0	0.485
**DBP (mmHg)**	77.5±11.5	75.9±11.7	<.001	77.6±12.3	77.4±12.3	0.417
**Heart rate (bpm)**	83.3±13.5	82.5±13.6	<.001	83.2±13.5	83.0±13.5	0.460
**HbA1c (%)**	8.1±1.4	7.5±1.2	<.001	8.0±1.6	7.2±1.1	<.001
**Triglycerides (mg/dL)**	159.4±112.4	149.2±119.2	<.001	156.9±123.4	147.8±107.8	<.001
**LDL (mg/dL)**	92.6±29.8	91.7±29.8	0.045	92.6±37.0	88.7±28.4	<.001
**HDL (mg/dL)**	45.6±11.8	47.5±12.4	<.001	45.8±12.3	45.8±12.3	0.999

HbA1c, hemoglobin A1c; DBP, diastolic blood pressure; DPP4i, dipeptidyl peptidase-4 inhibitor; HDL, high density lipoprotein; HR, heart rate; LDL, low density lipoprotein; SBP, systolic blood pressure

Data are expressed as the mean ± standard deviation (SD), (med, IQR), or as percentage.

Supplemental Table S3

Risk of adverse kidney outcomes for the paired study cohorts receiving empagliflozin vs. dapagliflozin after PSM, according to different categories of past eGFR slope prior to drug treatment

	SGLT2i Empagliflozin Event-Rate (per 100 PYs)	SGLT2i Dapagliflozin Event-Rate (per 100 PYs)	aHR; [95CI]	*P for int.*
**MAKE**				
Overall	1.59	1.70	0.85; [0.63-1.13]	
Past eGFR increase > 0 ml/min/1.73 m^2/year	1.31	1.34	0.72; [0.46-1.25]	
Past eGFR decrease 0-5 ml/min/1.73 m^2/year	1.07	1.28	0.84; [0.47-1.48]	0.95
Past eGFR decrease ≥ 5 ml/min/1.73 m^2/year	3.06	3.09	0.98; [0.60-1.61]	
**Abrupt decline in kidney function**				
Overall	1.08	0.89	1.04; [0.73-1.49]	
Past eGFR increase > 0 ml/min/1.73 m^2/year	1.21	0.81	1.22; [0.66-2.24]	
Past eGFR decrease 0-5 ml/min/1.73 m^2/year	0.82	0.74	1.15; [0.59-2.23]	0.53
Past eGFR decrease ≥ 5 ml/min/1.73 m^2/year	1.36	1.30	0.95; [0.48-1.89]	
**Post-treatment eGFR decrease ≥ 5 ml/min/1.73 m^2/year***				
Overall	16.20	14.39	1.06; [0.90-1.24]	
Past eGFR increase > 0 ml/min/1.73 m^2/year	16.37	12.48	1.21; [0.92-1.60]	
Past eGFR decrease 0-5 ml/min/1.73 m^2/year	14.25	12.95	1.14; [0.87-1.49]	0.15
Past eGFR decrease ≥ 5 ml/min/1.73 m^2/year	19.43	19.59	0.89; [0.66-1.21]	
**Deterioration in UACR***				
Overall	12.49	13.00	0.95; [0.80-1.13]	
Past eGFR increase > 0 ml/min/1.73 m^2/year	13.27	12.80	1.03; [0.77-1.39]	
Past eGFR decrease 0-5 ml/min/1.73 m^2/year	11.30	12.55	0.87; [0.66-1.15]	0.64
Past eGFR decrease ≥ 5 ml/min/1.73 m^2/year	13.47	14.06	1.00; [0.70-1.43]	

aHR = adjusted hazard ratio; CI = confidence interval; DPP4i = dipeptidyl peptidase-4 inhibitor; eGFR = estimated glomerular filtration rate; MAKE = major adverse renal event; PSM = propensity score matching; SGLT2i = sodium glucose cotransporter 2 inhibitor; T2D = type 2 diabetes; UACR = urine albumin-to-creatinine ratio

* Post-treatment eGFR decrease ≥5 mL/min/1.73 m² per year and deterioration in UACR are expressed as event-rate per 100 patients.

# Risk of outcomes was adjusted for age, gender, duration of diabetes, all baseline comorbidities, baseline body weight, HbA1c, eGFR, UACR, lipid profiles, ALT, systolic blood pressure, heart rate, all baseline cardiovascular drugs and anti-hyperglycemic agents in **Table 1**.

Supplemental Table S4

Risk of adverse kidney outcomes for the paired study cohorts receiving SGTL2i vs. DPP4i after PSM, according to different categories of past eGFR slope prior to drug treatment considering adjusting ALT as the covariate factor (Sensitivity analysis)

	**SGLT2i Event-Rate (per 100 PYs)**	**DPP4i Event-Rate (per 100 PYs)**	**aHR; [95CI]**	***P for int.***
**MAKE**				
Overall	1.68	2.81	0.79; [0.65-0.97]	
Past eGFR increase > 0 ml/min/1.73 m^2/year	1.37	2.29	0.67; [0.46-0.98]	0.94
Past eGFR decrease 0-5 ml/min/1.73 m^2/year	1.18	1.81	0.84; [0.58-1.21]	
Past eGFR decrease ≥ 5 ml/min/1.73 m^2/year	3.14	5.40	0.87; [0.63-1.19]	
**Abrupt decline in kidney function**				
Overall	1.06	1.50	0.76; [0.60-0.97]	
Past eGFR increase > 0 ml/min/1.73 m^2/year	1.10	1.49	0.80; [0.52-1.23]	0.97
Past eGFR decrease 0-5 ml/min/1.73 m^2/year	0.81	1.13	0.67; [0.43-1.04]	
Past eGFR decrease ≥ 5 ml/min/1.73 m^2/year	1.48	2.18	0.70; [0.45-1.07]	
**Post-treatment eGFR decrease ≥ 5 ml/min/1.73 m^2/year***				
Overall	15.28	20.59	0.73; [0.66-0.81]	
Past eGFR increase > 0 ml/min/1.73 m^2/year	14.67	19.61	0.74; [0.61-0.90]	0.98
Past eGFR decrease 0-5 ml/min/1.73 m^2/year	13.33	17.92	0.72; [0.60-0.85]	
Past eGFR decrease ≥ 5 ml/min/1.73 m^2/year	19.65	26.57	0.72; [0.60-0.88]	
**Deterioration in UACR***				
Overall	12.80	14.07	0.91; [0.81-1.03]	
Past eGFR increase > 0 ml/min/1.73 m^2/year	13.26	14.78	0.92; [0.74-1.14]	0.73
Past eGFR decrease 0-5 ml/min/1.73 m^2/year	11.98	12.26	0.96; [0.79-1.16]	
Past eGFR decrease ≥ 5 ml/min/1.73 m^2/year	13.57	16.53	0.84; [0.66-1.06]	

aHR = adjusted hazard ratio; CI = confidence interval; DPP4i = dipeptidyl peptidase-4 inhibitor; eGFR = estimated glomerular filtration rate; MAKE = major adverse renal event; PSM = propensity score matching; SGLT2i = sodium glucose cotransporter 2 inhibitor; T2D = type 2 diabetes; UACR = urine albumin-to-creatinine ratio

* Post-treatment eGFR decrease ≥5 mL/min/1.73 m² per year and deterioration in UACR are expressed as event-rate per 100 patients.

# Risk of outcomes was adjusted for age, gender, duration of diabetes, all baseline comorbidities, baseline body weight, HbA1c, eGFR, UACR, lipid profiles, ALT, systolic blood pressure, heart rate, all baseline cardiovascular drugs and anti-hyperglycemic agents in **Table 1**.

Supplemental Table S5

Risk of adverse kidney outcomes for the paired study cohorts receiving SGTL2i vs. DPP4i after PSM, according to different categories of past eGFR slope prior to drug treatment specifically focused on empagliflozin (Sensitivity analysis)

	**SGLT2i Event-Rate (per 100 PYs)**	**DPP4i Event-Rate (per 100 PYs)**	**aHR; [95CI]**	***P for int.***
**MAKE**				
Overall	1.59	2.81	0.73; [0.57-0.93]	
Past eGFR increase > 0 ml/min/1.73 m^2/year	1.31	2.24	0.54; [0.34-0.88]	0.80
Past eGFR decrease 0-5 ml/min/1.73 m^2/year	1.07	1.81	0.73; [0.45-1.17]	
Past eGFR decrease ≥ 5 ml/min/1.73 m^2/year	3.06	5.43	0.87; [0.58-1.32]	
**Abrupt decline in kidney function**				
Overall	1.08	1.47	0.77; [0.57-1.04]	
Past eGFR increase > 0 ml/min/1.73 m^2/year	1.21	1.44	0.80; [0.47-1.35]	0.83
Past eGFR decrease 0-5 ml/min/1.73 m^2/year	0.82	1.10	0.73; [0.42-1.26]	
Past eGFR decrease ≥ 5 ml/min/1.73 m^2/year	1.36	2.19	0.60; [0.34-1.06]	
**Post-treatment eGFR decrease ≥ 5**				
Overall	16.20	20.54	0.77; [0.68-0.88]	
Past eGFR increase > 0 ml/min/1.73 m^2/year	16.37	19.66	0.82; [0.65-1.03]	0.70
Past eGFR decrease 0-5 ml/min/1.73 m^2/year	14.25	17.82	0.77; [0.62-0.95]	
Past eGFR decrease ≥ 5 ml/min/1.73 m^2/year	19.43	26.45	0.71; [0.55-0.90]	
**Deterioration in UACR**				
Overall	12.49	14.09	0.90; [0.77-1.04]	
Past eGFR increase > 0 ml/min/1.73 m^2/year	13.27	14.87	0.92; [0.71-1.20]	0.93
Past eGFR decrease 0-5 ml/min/1.73 m^2/year	11.30	12.23	0.91; [0.71-1.16]	
Past eGFR decrease ≥ 5 ml/min/1.73 m^2/year	13.47	16.52	0.85; [0.63-1.15]	

The abbreviations as in **Supplemental Table S3.**

* Post-treatment eGFR decrease ≥5 mL/min/1.73 m^2 per year and deterioration in UACR are expressed as event-rate per 100 patients.

# Risk of outcomes was adjusted for age, gender, duration of diabetes, all baseline comorbidities, baseline body weight, HbA1c, eGFR, UACR, lipid profiles, systolic blood pressure, heart rate, all baseline cardiovascular drugs and anti-hyperglycemic agents in **Table 1**.

Supplemental Table S6

Risk of adverse kidney outcomes for the paired study cohorts receiving SGTL2i vs. DPP4i after PSM, according to different categories of past eGFR slope prior to drug treatment specifically focused on dapagliflozin (Sensitivity analysis)

	**SGLT2i Event-Rate (per 100 PYs)**	**DPP4i Event-Rate (per 100 PYs)**	**aHR; [95CI]**	***P for int.***
**MAKE**				
Overall	1.70	2.81	0.81; [0.63-1.04]	
Past eGFR increase > 0 ml/min/1.73 m^2/year	1.34	2.24	0.69; [0.43-1.12]	0.94
Past eGFR decrease 0-5 ml/min/1.73 m^2/year	1.28	1.81	0.93; [0.59-1.47]	
Past eGFR decrease ≥ 5 ml/min/1.73 m^2/year	3.09	5.43	0.79; [0.53-1.17]	
**Abrupt decline in kidney function**				
Overall	0.89	1.47	0.63; [0.45-0.88]	
Past eGFR increase > 0 ml/min/1.73 m^2/year	0.81	1.44	0.59; [0.32-1.08]	0.89
Past eGFR decrease 0-5 ml/min/1.73 m^2/year	0.74	1.10	0.59; [0.33-1.06]	
Past eGFR decrease ≥ 5 ml/min/1.73 m^2/year	1.30	2.19	0.57; [0.32-1.04]	
**Post-treatment eGFR decrease ≥ 5**				
Overall	14.39	20.54	0.69; [0.60-0.80]	
Past eGFR increase > 0 ml/min/1.73 m^2/year	12.48	19.66	0.63; [0.49-0.82]	0.58
Past eGFR decrease 0-5 ml/min/1.73 m^2/year	12.95	17.82	0.70; [0.56-0.88]	
Past eGFR decrease ≥ 5 ml/min/1.73 m^2/year	19.59	26.45	0.72; [0.56-0.92]	
**Deterioration in UACR**				
Overall	13.00	14.09	0.94; [0.80-1.09]	
Past eGFR increase > 0 ml/min/1.73 m^2/year	12.80	14.87	0.88; [0.67-1.15]	0.63
Past eGFR decrease 0-5 ml/min/1.73 m^2/year	12.55	12.23	1.03; [0.80-1.31]	
Past eGFR decrease ≥ 5 ml/min/1.73 m^2/year	14.06	16.52	0.86; [0.64-1.15]	

The abbreviations as in **Supplemental Table S3.**

* Post-treatment eGFR decrease ≥5 mL/min/1.73 m² per year and deterioration in UACR are expressed as event-rate per 100 patients.

\# The adjusted factors as in **Supplemental Table S4.**

Supplemental Table S7

Risk of adverse kidney outcomes for the paired study cohorts receiving SGTL2i vs. DPP4i after PSM, according to different categories of past eGFR slope prior to drug treatment according to different definition of MAKE (Sensitivity analysis)

	**SGLT2i Event-Rate (per 100 PYs)**	**DPP4i Event-Rate (per 100 PYs)**	**aHR; [95CI]**	***P for int.***
**MAKE (> 50% reduction in follow-up eGFR or the development of ESKD)**				
Overall	1.67	2.81	0.77; [0.64-0.94]	
Past eGFR increase > 0 ml/min/1.73 m^2/year	1.35	2.24	0.66; [0.45-0.96]	0.91
Past eGFR decrease 0-5 ml/min/1.73 m^2/year	1.18	1.81	0.82; [0.57-1.19]	
Past eGFR decrease ≥ 5 ml/min/1.73 m^2/year	3.11	5.43	0.87; [0.64-1.19]	
**MAKE (> 40% reduction in follow-up eGFR or the development of ESKD)**				
Overall	2.65	4.29	0.75; [0.64-0.87]	
Past eGFR increase > 0 ml/min/1.73 m^2/year	2.07	3.85	0.58; [0.43-0.78]	0.18
Past eGFR decrease 0-5 ml/min/1.73 m^2/year	1.98	2.87	0.76; [0.58-1.01]	
Past eGFR decrease ≥ 5 ml/min/1.73 m^2/year	4.94	7.63	0.90; [0.70-1.16]	
**MAKE (> 30% reduction in follow-up eGFR or the development of ESKD)**				
Overall	4.95	6.68	0.88; [0.78-0.99]	
Past eGFR increase > 0 ml/min/1.73 m^2/year	4.77	6.57	0.79; [0.64-0.97]	0.47
Past eGFR decrease 0-5 ml/min/1.73 m^2/year	3.71	4.76	0.88; [0.71-1.08]	
Past eGFR decrease ≥ 5 ml/min/1.73 m^2/year	7.72	10.60	0.97; [0.79-1.19]	
**MAKE (> 60% reduction in follow-up eGFR or the development of ESKD)**				
Overall	1.05	2.15	0.70; [0.55-0.88]	
Past eGFR increase > 0 ml/min/1.73 m^2/year	0.73	1.44	0.57; [0.35-0.94]	0.86
Past eGFR decrease 0-5 ml/min/1.73 m^2/year	3.75	1.45	0.69; [0.44-1.07]	
Past eGFR decrease ≥ 5 ml/min/1.73 m^2/year	2.16	4.37	0.86; [0.59-1.23]	
**MAKE (> 2 folds in follow-up sCr or the development of ESKD)**				
Overall	1.27	2.42	0.69; [0.56-0.85]	
Past eGFR increase > 0 ml/min/1.73 m^2/year	0.98	1.80	0.58; [0.37-0.89]	0.96
Past eGFR decrease 0-5 ml/min/1.73 m^2/year	0.91	1.61	0.68; [0.45-1.02]	
Past eGFR decrease ≥ 5 ml/min/1.73 m^2/year	2.43	4.37	0.80; [0.57-1.13]	

ESKD = end stage kidney disease

Other abbreviations as in **Supplemental Table S3.**

* Post-treatment eGFR decrease ≥5 mL/min/1.73 m^2 per year and deterioration in UACR are expressed as event-rate per 100 patients.

The adjusted factors as in **Supplemental Table S4.**
